# Prevalence and predictors of exclusive breastfeeding among women in Kigoma region, Western Tanzania: a community based cross-sectional study

**DOI:** 10.1186/1746-4358-6-17

**Published:** 2011-11-09

**Authors:** Tiras Eshton Nkala, Sia Emmanueli Msuya

**Affiliations:** 1 Department of Community Health, Tumaini University, KCM College, Box 2240, Moshi, Tanzania; 2 KCMC, Moshi, Tanzania

**Keywords:** Exclusive breastfeeding, mixed feeding, prevalence, predictors, Tanzania

## Abstract

**Background:**

Exclusive breastfeeding (EBF) for the first six months of infants' lives is a cost effective intervention in saving children's lives and can avert 13 - 15% of the 9 million deaths of children under 5 years old in resource poor settings. However, EBF rates have been shown to be low in resource poor settings, ranging between 20 and 40%. In Tanzania, the prevalence of EBF among infants under 6 months is 41%, with limited information on predictors of EBF. The aim of the study was to determine prevalence of EBF and its predictors in Kigoma Municipality, Western Tanzania.

**Methods:**

A cross-sectional study was conducted in March to May 2010 among 402 consenting women, with infants aged 6 to 12 months, from randomly selected households. A questionnaire was used to collect information on demographic characteristics, knowledge of EBF, infant feeding practices, and on HIV status.

**Results:**

The prevalence of EBF among women in Kigoma Municipality was 58%. Knowledge of EBF was relatively higher (86%) compared to the practice. In the multivariable analysis, women with adequate knowledge of EBF (AOR 5.4), women who delivered at health facilities (AOR 3.0) and women who had no problems related to breasts, like engorgement/cracked nipples (AOR 6.6) were more likely to exclusively breastfeed compared to others.

**Conclusions:**

Prevalence of EBF in Kigoma municipality was slightly higher than the national figure of 41%, however it was way below the EBF prevalence of 90% recommended by the WHO. Strategies that target improving knowledge and skills for lactation management among women, as well as strategies to improve health facility delivery, may help to improve EBF in this setting.

## Background

Exclusive breastfeeding (EBF) for the first six months of an infant's life is a cost effective intervention in saving children's lives and it is recommended by the World Health Organisation (WHO) [1]. EBF means giving only breast milk to the infant, without mixing it with water, other liquids, tea, herbal preparations or food in the first six months of life, with the exception of vitamins, mineral supplements or medicines [1]. It is estimated that, with EBF coverage of 90%, 13 to 15% of deaths of children under 5 years could be averted in low and middle income countries [2]. Exclusively breastfed infants have been shown to have lower rates of acute respiratory infections and diarrhoea, to have better neuro-developmental outcomes and have better physical growth compared to mix-fed or non-breastfed infants [1-6]. In areas where HIV prevalence is high, especially in sub-Saharan Africa, EBF has been shown to have an added advantage of reducing the rates of mother-to-child transmission of HIV (MTCT) [7-9].

The prevalence of EBF, however, is low globally (39%), and it is estimated to be 36% in low income countries [1,10]. Despite reports of increased EBF in most areas (22% to 30% in sub-Saharan Africa, and 30% to 45% in Latin America and the Caribbean, excluding Brazil, and Mexico) the prevalence is still low compared to the WHO recommendation [1,10]. Child mortality is high in these low and middle income countries where EBF prevalence is low. In Tanzania, for example, mortality rates of infant and children younger than five years are high (infant mortality rate 51 per 1, 000 live births and death rate of children younger than 5 years is 81 per 1000 live births respectively) while the prevalence of EBF among infants below 6 months is low (41%) [11,12]. Hence, understanding the factors that influence EBF is essential to help in the development of strategies to promote EBF practices.

Predictors of EBF vary widely between and within countries. Urban or rural difference, age, employment status, higher education, knowledge about good breastfeeding practices, positive attitudes towards EBF, intent to exclusively breastfeed before delivery, partner living with the woman, mode of delivery, birth weight of the infant, health system practices and community beliefs have all been shown to influence the prevalence of EBF in different areas [13-16]. Information on the prevalence, and on factors influencing EBF is, however, limited in Tanzania and relatively old [11,16]. This study, thus, aimed to determine the prevalence of EBF and its predictors among women in Kigoma municipality, Western Tanzania.

## Methods

### Study area

A community-based cross sectional study was conducted between March and May 2010 among women with infants aged 6 - 12 months in Kigoma Municipality. The municipality is one of the four districts of Kigoma region, situated in Western Tanzania, on the shores of Lake Tanganyika. Other districts are Kibondo, Kasulu and Kigoma rural [17].

The region consists mainly of a rural population and the main economic activities include agriculture, livestock keeping, fishing and petty business. The region has a population of 1, 674, 046 of whom, 144, 256 live in Kigoma Municipality. The municipality has approximately 68, 813 women of child bearing age and 11, 542 infants. Reproductive and child health (RCH) services including antenatal care, postnatal care and immunizations are available in ten dispensaries, two health centres and one hospital (DMO: District Health Annual Report, Kigoma 2009). It is estimated that 92% of pregnant women attend at least once for antenatal care, 39% of the women deliver at health facilities and about 80% of the children below one year receive complete immunization [11,18]

### Sampling procedure

Administratively, the Kigoma Municipality, like other districts in Tanzania is divided into divisions, wards and finally into the hamlets (*mitaa*) in urban areas or villages in the rural areas. The hamlets are the smallest units consisting of about 20 to 40 households with a leader. The municipality has 2 divisions, 13 wards and a total of 63 hamlets (*mitaa*).

Multi-stage sampling technique was used to select the participants. The first stage involved a random selection of 4 wards out of 13 in Kigoma Municipality. Then 6 hamlets were randomly selected from each of the 4 selected wards, giving a total of 24 hamlets. All the households in the 24 selected hamlets with women who met the inclusion criteria were eligible to participate in the study. Women were listed with the help of community leaders who had local statistics regarding their areas.

All the women from the households with infants aged 6-12 months were invited to participate in the study. Out of the 408 women listed and approached, 402 (98.5%) agreed to participate in the study.

### Interviews

Women were first informed about the study and its aim, and those agreeing to participate gave a written consent. Face-to-face interviews were then conducted at participant's home, at a private spot away from other family members, in order to understand each other and for confidentiality. A standardized questionnaire was used to collect information on the socio-demographic characteristics of the women and their partners, on knowledge of EBF, on cultural beliefs regarding breastfeeding and EBF, on infant feeding practices using a "recall since birth" method, advice on EBF given during pregnancy, advice given during post partum period as well as on history of HIV counselling and testing during pregnancy. After the interviews, infants were weighed using an infant weighing Salter Scales machine (in kilograms) and height measured (in centimetres) in prone using infant length boards.

### Data processing and analysis

Data were entered and analysed using Statistical Package for the Social Sciences (SPSS) software for windows version 13.0 and Epi Info 6 for Dos version 6.04d. Knowledge of EBF was assessed using two questions; meaning or definition of EBF and recommended duration of six months. Respondents who answered both questions correctly were categorized as having 'adequate knowledge' of EBF. Prevalence of exclusive breastfeeding was measured using questions on introduction of prelacteal feeding, age of introduction of liquids/semisolid or solid foods and breastfeeding duration of six months. Respondents, who scored three points i.e. did not give any prelacteal feeding, breastfed infants for six months and did not introduce any liquids/solids before six months were categorized to have practised "EBF". Others were categorised to have "mix fed" the infants.

Descriptive statistics were used to summarize the data. Difference between groups was tested using Pearson Chi-square test and Fisher's Exact test where required. The Odds ratio (OR) and their 95% confidence intervals (CI) were used to assess the strength of association between several factors and the prevalence of EBF. All of the predictor variables with p value of < 0.05 in the bivariate analysis were included in the regression model. Logistic regression was performed to get independent predictors for exclusive breastfeeding. A p-value of < 0.05 was taken as significant.

### Ethics

Ethics approval for the study was obtained from the KCM College ethics research committee (Certificate number 326/2010). Permission to conduct the study in the local area was obtained from the District Medical Officer and leaders of selected areas. Written consent was sought from every participant. For illiterate women, a right thumb print was taken as a signature.

## Results

A total of 402 women participated in the study. The mean age (standard deviation [SD]) of the women was 25.5 (± 5.6) years, range 15 to 48 and the median number of children was 2; range 1 to 9. The majority of the women were married/cohabiting (85%), had only primary education (75%), and were not employed (70%). Their partners' ages ranged from 19- 60 years with mean (SD) of 32 (± 6.9) years and 79% had primary education (see Table 1).

**Table 1:** Socio-demographic characteristics of respondents and partners (n = 402) and comparison with population data

Characteristics	Frequency	%	Population data*%
***Mother***			
**Age (years)**			
15-24	195	48.5	
25-49	207	51.5	
**Education**			
None	79	19.8	24.5
Primary	301	74.9	71.7
Secondary & above	22	5.5	3.8
**Employment**			
Employed	120	29.9	29.0
Not Employed	282	70.1	71.0
**Marital status**			
Single/divorced/widowed	59	14.7	32.7
Married/cohabiting	343	85.3	67.3
**Number of children**			
One child	97	24.1	
Above one child	305	75.9	
***Partner***			
Age in years (n = 378)**			
25 or less	61	16.1	
Above 25	317	83.9	
Education (n = 386)**			
None	28	7.3	
Primary	305	79.0	
Secondary & above	53	13.7	

**Some women did not know their partner's age and/or their education level.

*Tanzania Demographic and Health Survey 2004/05 [12]

Fifty seven percent of the respondents' children were female. Their mean age was 9.2 months (SD, ± 2.2); range 6-12 months. Nutritional status among children showed that 9.7% were underweight (Weight-for-Age z-score < -2 SD), 6.7% were wasted (< -2 SD Weight-for-Height z-score) and 16.4% were stunted (Height-for-Age z-score < -2 SD).

Table 2 depicts knowledge of the participants of exclusive breastfeeding. Eighty six percent of the participants had adequate knowledge on the definition/meaning of EBF. There was no association between several socio-demographic factors (age, education, income, marital status, parity or employment) with EBF knowledge. But women who reported to have had any pathological problems related to breasts, like engorgement, cracked nipples or mastitis in the first six months after delivery, were less likely to have adequate knowledge on EBF (75%) compared to women who did not report such problems (89%); [OR 0.38; (95% CI: 0.20, 0.73)].

**Table 2:** Knowledge of respondents about exclusive breastfeeding (n = 402)

Variable	Attribute	Score category	n	%
Meaning of 'Exclusive Breastfeeding'	Feed only breast milk	Correct	386	96.2
	Feed only cow's milk	Incorrect	5	1.2
	Feed only formula milk	Incorrect	7	1.7
	Feed breast milk and other foods	Incorrect	3	0.7
	Don't know	Incorrect	1	0.2
Recommended duration of exclusive breastfeeding	From birth to six months	Correct	361	89.8
	From birth to four months	Incorrect	23	5.7
	From birth to three months	Incorrect	18	4.5
Knowledge (Overall)	Adequate knowledge	Correct in both	347	86.3
	Inadequate knowledge	Incorrect in either	55	13.7

Most women (366, 91%) reported to have breastfed their infants within one hour after delivery. Prelacteal feeds such as water, glucose, juice, soda, formula, cow's milk and porridge were given by 88 women (22%). Fifty eight percent (232) of the 402 respondents reported having exclusively breastfed their infants for 6 months. Table 3 shows the association between EBF and socio-demographic characteristics of the participants as well as health service related factors. Women had higher odds of exclusive breastfeeding if they were; employed (p = 0.005), had adequate knowledge of EBF (p < 0.001), had delivered at a health facility (p < 0.001), and had partners with secondary or higher education (p = 0.002). Women who reported to have had pathological problems during the first 6 months were 86% less likely to have exclusively breastfed their infants than women who did not encounter such problems; (OR: 0.14; 95% CI 0.07, 0.26).

**Table 3:** Association between exclusive breastfeeding and socio-demographic and health service related factors among 402 respondents

	Exclusively breastfed (n = 232)				
				
Variable	Total (N)	n	(%)	OR (95%CI)	p value
***Mother***					
**Age (year)**					
15-24	195	120	61.5	1	
25-49	207	112	54.1	14 (0.9, 2.0)	0.132
**Education**					
None/Primary	380	218	57.4	1	
Secondary & above	22	14	63.6	1.3 (0.5, 3.5)	0.563
**Employment**					
Not Employed	282	150	53.2	1	
Employed	120	82	68.3	1.9 (1.2, 3.0)	0.005
**Marital status**					
Married	343	191	55.7	1	
Single	59	41	69.5	1.8 (1.0, 3.3)	0.047
**Number of children**					
> one child	305	173	56.7	1	
One child	97	59	60.8	1.2 (0.7, 1.9)	0.476
**Knowledge of EBF**					
Inadequate	55	12	21.8	1	
Adequate	347	220	63.4	6.2 (3.2, 12.2)	< 0.001
**Advised about EBF during pregnancy**					
Yes	206	113	54.8	1	
No	196	119	60.7	1.3 (0.9, 1.9)	0.967
**Place of delivery**					
Home	148	61	41.2	1	
Health facility	254	171	67.3	2.9 (1.9, 4.6)	< 0.001
Pathology problems*					
Yes	68	14	20.6	1	
No	334	218	65.3	7.3 (3.9, 13.6)	< 0.001
**HIV status**^**†**^					
Positive	6	3	50.0	1	
Negative	358	211	58.9	1.4 (0.2, 9.0)	0.694
***Partner***					
Age in year (n = 378)**					
Above 25	317	180	56.8	1	
25 or less	61	35	57.4	1.0 (0.6, 1.8)	0.932
Education (n = 386)**					
None/primary Secondary	333	181	54.4	1	
& above	53	41	77.4	2.87 (1.40, 6.00)	0.002
***Children's information***					
**Underweight (W/A)**					
Normal	363	212	58.4	1	
Underweight	39	20	51.3	1.33 (0.69, 2.59)	0.392
**Stunting (H/A)**					
Normal	336	200	59.5	1	
Stunted	66	32	48.5	1.56 (0.92, 2.56)	0.097

**^†^**Some ANC cards could not be found

*Problems related to mothers' breasts (engorgement, sore/cracked nipple, mastitis or abscess)

**Some women did not know their partner's age and/or their education level.

In the logistic regression analysis, EBF remained independently associated with health facility delivery (adjusted odds ratio [AOR] = 3.0), adequate knowledge of EBF (AOR = 5.4) and report of absence of pathological breast problems (AOR = 6.6); see Table 4.

**Table 4:** Logistic regression of the predictors of exclusive breastfeeding

Variable	**AoR**^**†**^	95%CI	p value
Knowledge about EBF			
Inadequate knowledge of EBF	Reference		
Adequate knowledge of EBF	5.4	2.5, 11.6	< 0.001
Pathological problems*			
Yes	Reference		
No	6.6	3.2, 13.6	< 0.001
Place of delivery			
Home	Reference		
Health facility	3.0	1.7, 5.4	< 0.001

AoR Adjusted Odds Ratio

**^† ^**Variables included in the model: employment, marital status, place of delivery, partner's education, pathological problems and knowledge of EBF

*Problems related to mothers' breasts (engorgement, sore/cracked nipple, mastitis or abscess).

## Discussion

The study results showed that the proportion of children who were exclusively breastfed for 6 months was 58% in Kigoma Municipality, with both health-related factors and knowledge influencing the practice. The study also showed that approximately 14% of the women with infants aged 6 - 12 months had inadequate knowledge of EBF.

The prevalence of EBF was 58%. Though higher than the national average of 41% and than the rate reported for Morogoro Tanzania in late 90 s [11,16], the 58% prevalence rate of exclusive breastfeeding for 6 months found in this study is still well below the 90% level recommended by the WHO [1,2]. It was however, higher than the EBF prevalence reported from Zimbabwe (7%), Switzerland (38%), Norway (44%), Bauru, Brazil (24.2%) and Nigeria (27%) respectively [9,13,15,19,20]. These results show wide variation of EBF prevalence between and within countries and over time. Different methodologies for estimating the rate of EBF may also influence the results [21]. Exclusive breastfeeding is a cost-effective intervention in saving infants' live, however the coverage of 90% has to be reached to benefit from this intervention [1,2]. Therefore there is a need to intensify community and hospital-based interventions which promote EBF in Kigoma Municipality.

In this study, 17% of the women reported to have had breast problems during breastfeeding, such as engorged breasts, cracked nipples and mastitis. These women were significantly less likely to exclusively breastfeed and significantly less likely to have adequate knowledge of EBF. Incorrect attachment and infrequent feeding of babies during breastfeeding are the main causes of breast problems [1]. Studies in Brazil and in urban & rural Tanzania respectively have also observed that women with breast problems when breastfeeding tend to mix feed early. In the Tanzanian study researchers found that 95% of the women with breast problems did not know what to do when the problems developed and they had low skills on how to attach and support the infants [6,16]. Both in Brazil and Tanzania when women had breastfeeding problems they believed that the quality of milk was affected or it was insufficient, and this is why they introduced other foods [6,16]. Health education during pregnancy and immediately post delivery on practical issues/skills like positioning and attachment of the baby during BF is important in Kigoma. Further, women should be educated on what to do and where to seek care if breast problem arises.

In the present study, adequate knowledge of EBF influenced the prevalence of EBF; the higher the level of adequate knowledge of EBF among women, the higher the prevalence of EBF (63% vs. 22%). Women's high knowledge may be due to a combination of factors; policies supporting breastfeeding, the Baby Friendly Hospital Initiative (BFHI), which has been strongly promoted in the country since the 1990s, community education on EBF to women in general, and increased support for EBF among women, especially since 2004 when the PMTCT program was introduced and scaled up in the country. Despite the efforts, 14% of the women had inadequate knowledge of EBF. These women tended to give prelacteal feeds and introduced complementary feeding early to infants aged less than six months. One study in Australia has shown that mothers who were aware and had knowledge of the WHO EBF recommendations were about five times more likely to intend to breastfeed exclusively compared to those without EBF awareness [22]. Intensified efforts are needed to make sure that women have universal access to current information regarding EBF and its advantages.

Women delivering at the health facilities had three times the odds of reporting practicing EBF compared to those who delivered at home. Results are similar to those observed in Switzerland, Italy and Brazil, where hospital delivery and health facility factors like rooming-in and nursing guidance at maternity hospitals were strong determinants for EBF, even more than socio-demographic factors [13,23,24]. The Tanzania Demographic and Health Survey (TDHS) 2004/05 report showed that, compared to home deliveries, children of mothers assisted during delivery by health care personnel at health facilities were more likely to be breastfed within one hour after birth (66% compared to 55%), were less likely to be given prelacteal feeds (30% vs. 41%) and were more likely to have higher EBF prevalence [11]. Availability of policy, breastfeeding guidelines, and training for most staff on EBF in the era of PMTCT might have contributed to higher EBF knowledge and counselling skills among health workers. This in turn might have led to women getting support and help with breast feeding issue early and immediately after delivery. Probably during this time the health workers also reinforce the idea of EBF and its importance.

Hospital-based breastfeeding promotion efforts might be cost effective to implement in Tanzania. Antenatal care is widely attended by 96% of pregnant women, out of whom 94% are attended by trained health personnel both in rural and urban areas [11,12]. This opportunity should be used to intensify efforts at informing women about the importance of breastfeeding, especially EBF. Printed materials with simple message (brochure & pictures), and mass media can also be used, as it has shown to increase knowledge and positive attitudes towards EBF elsewhere [24,25].

Breastfeeding promotion interventions immediately after delivery have shown to have a strong effect on EBF prevalence in many studies [13,23-26].

At health facilities in Tanzania, breastfeeding guidelines require that health workers should help women to initiate breastfeeding immediately after delivery. They should also support women practically, with issues of attachment and how to hold the baby during breastfeeding [26].

It may be that practical demonstrations and information on EBF given immediately after delivery make more sense to women and are easier to follow than information given during pregnancy [19,24-26]. Promotion of health facility delivery in Tanzania, which is still low (47%, and 39% in Kigoma region), will not only benefit breastfeeding efforts, but may also help to improve other maternal and neonatal outcomes [11,12].

The study has several limitations which have to be considered. The major limitation may be recall bias. We recruited women with infants aged 6-12 months and asked them about feeding practices of the infants when they were less than 6 months of age. This required a long recall period and some women might have forgotten the time when liquids including water or semi-solids were introduced and given wrong accounts. This might bias our estimate of EBF towards a higher value i.e. some who predominantly breastfed to be classified as EBF. Data on EBF also relied on self report by the women. Given the high awareness on EBF in this setting (86%), reporting bias may have also influenced the results, i.e. women reporting what is known as good breastfeeding practice and not the actual practice. This will also bias the EBF estimate towards a higher value. We did not evaluate the health facilities to observe if they are advising and supporting women to exclusively breastfeed, and if they are following the BFHI guidelines. Measuring EBF prevalence with a cross sectional study using recall since birth is difficult and inaccurate; a better design, which would have given a reliable estimate, would have been a longitudinal study, where a cohort of infants is followed from birth with frequent recordings of BF [7,21]. Despite the limitations, the women who participated in this study were a fair representation of the women of reproductive age in Kigoma, because their demographic characteristics were similar to the regional TDHS data; see population data in Table 1[12].

## Conclusion

In conclusion, though knowledge of EBF was high (86%) among women of reproductive age in Kigoma municipality, this was not matched with the prevalence of EBF (58%). The report of breast problems, adequate knowledge of EBF, and delivery at health facilities influenced the prevalence of EBF. Comprehensive education on breast problems and management, as well as education on positioning and attachment, should be offered to women during pregnancy and immediately after delivery in this setting. These should be complemented with community-based EBF support programs where possible. Qualitative studies are required in this setting to explore if the prevalence of EBF reported, which is higher than the national average, actually matches early infant feeding practices in the area.

## Competing interests

The authors declare that they have no competing interests.

## Authors' contributions

TEN and SEM conceived, developed and conducted the study. Both authors prepared and revised the manuscript and approved it.
